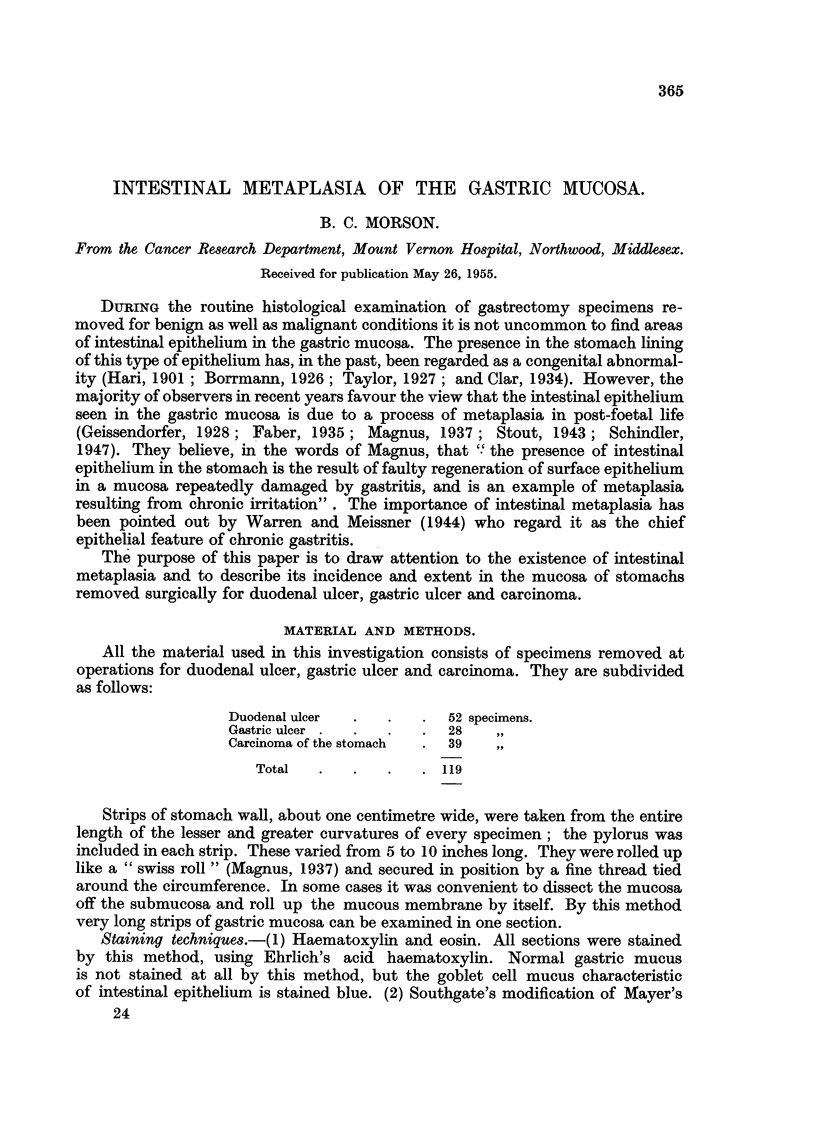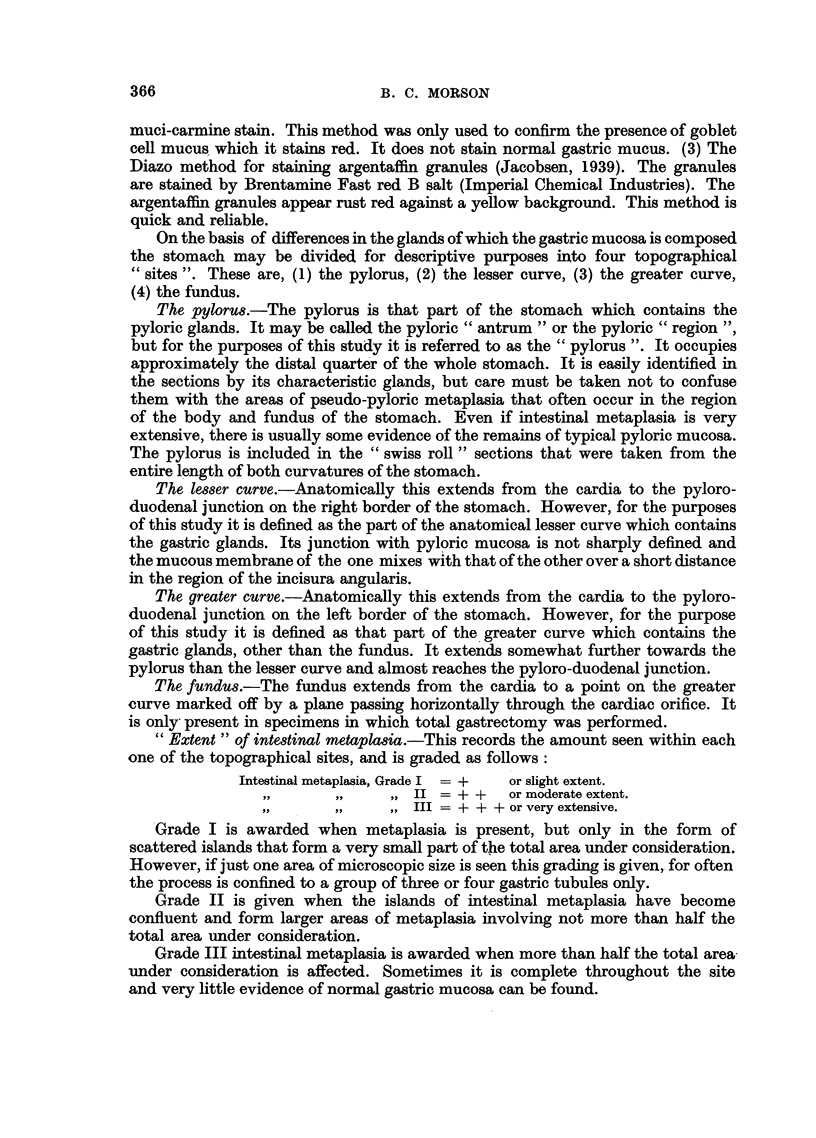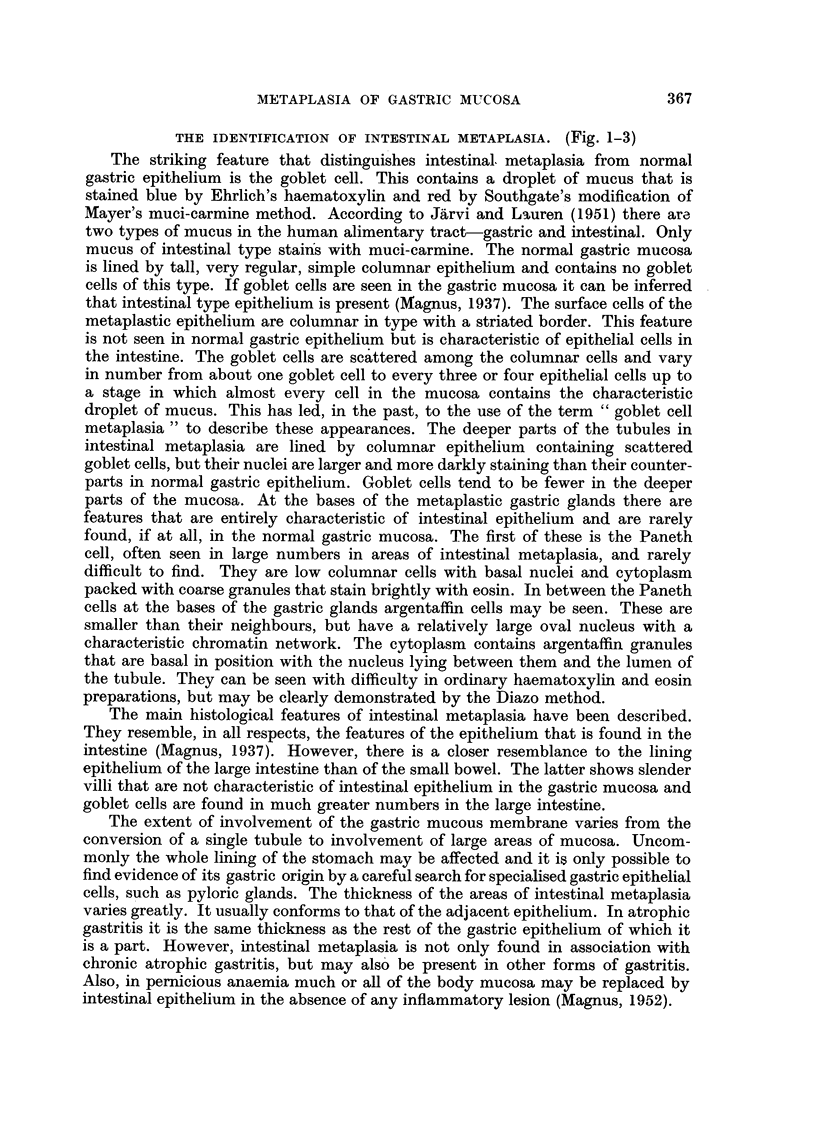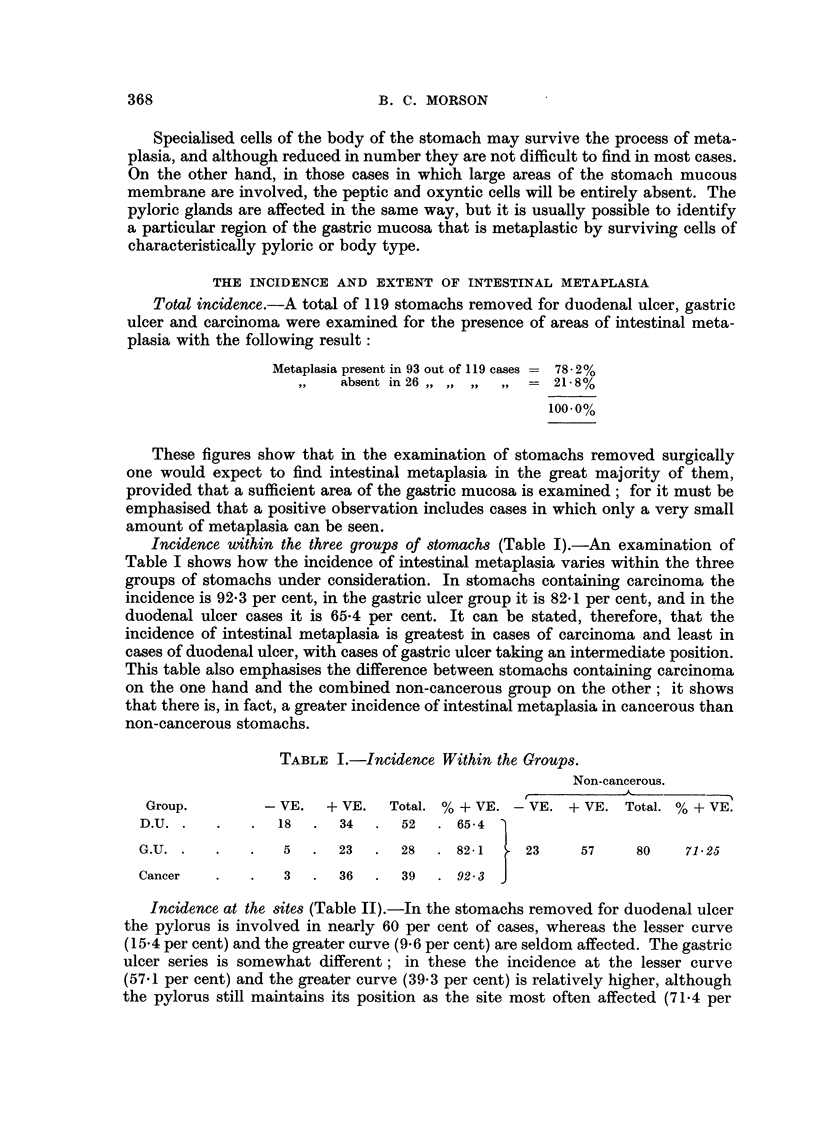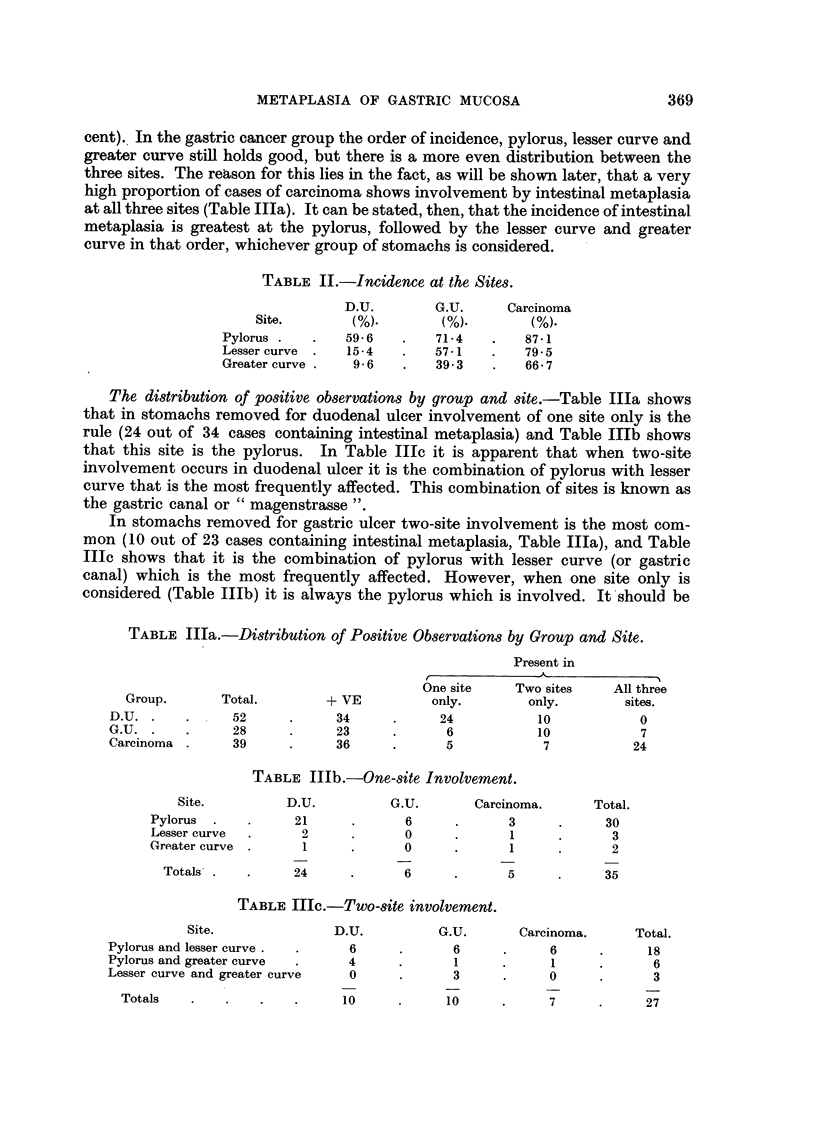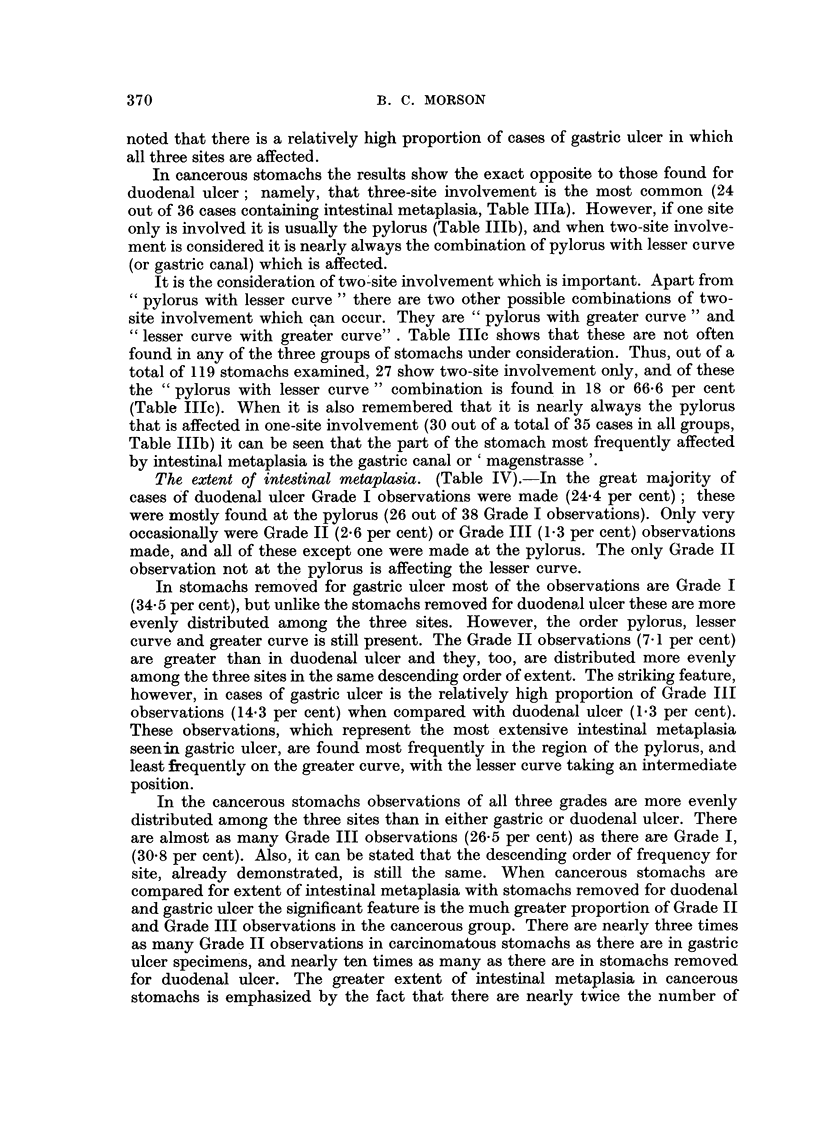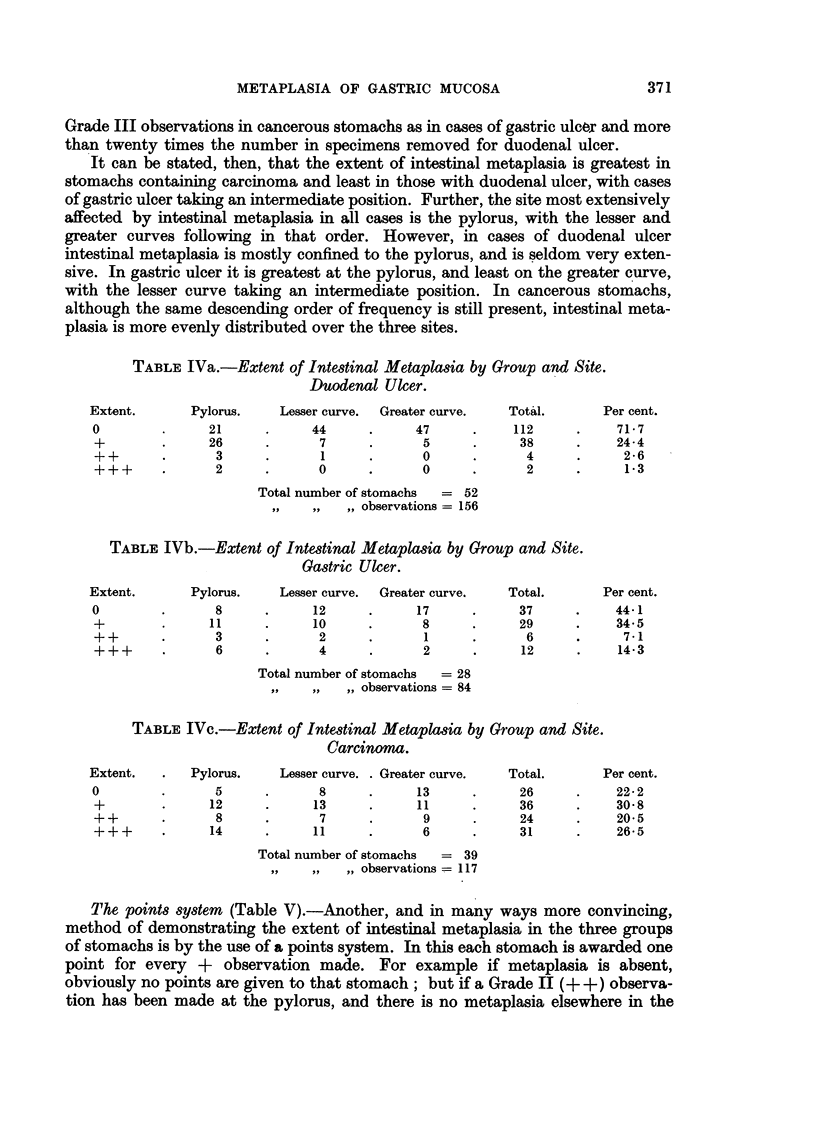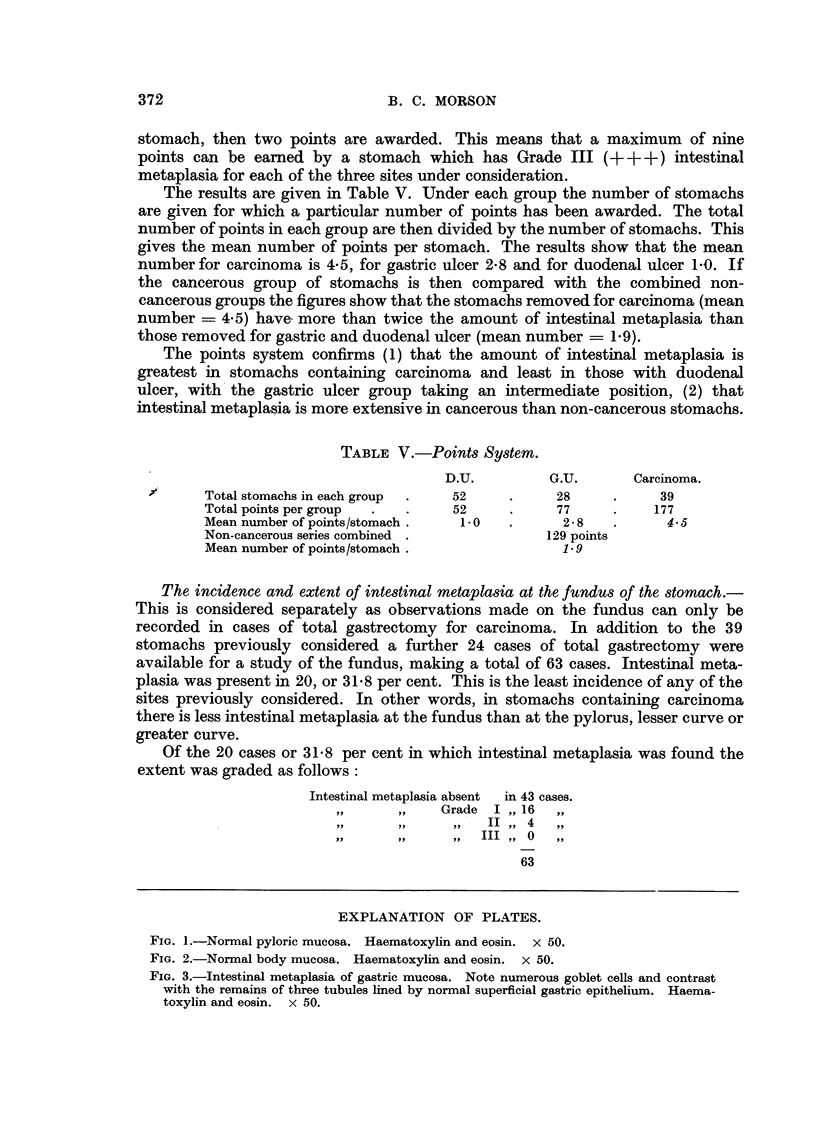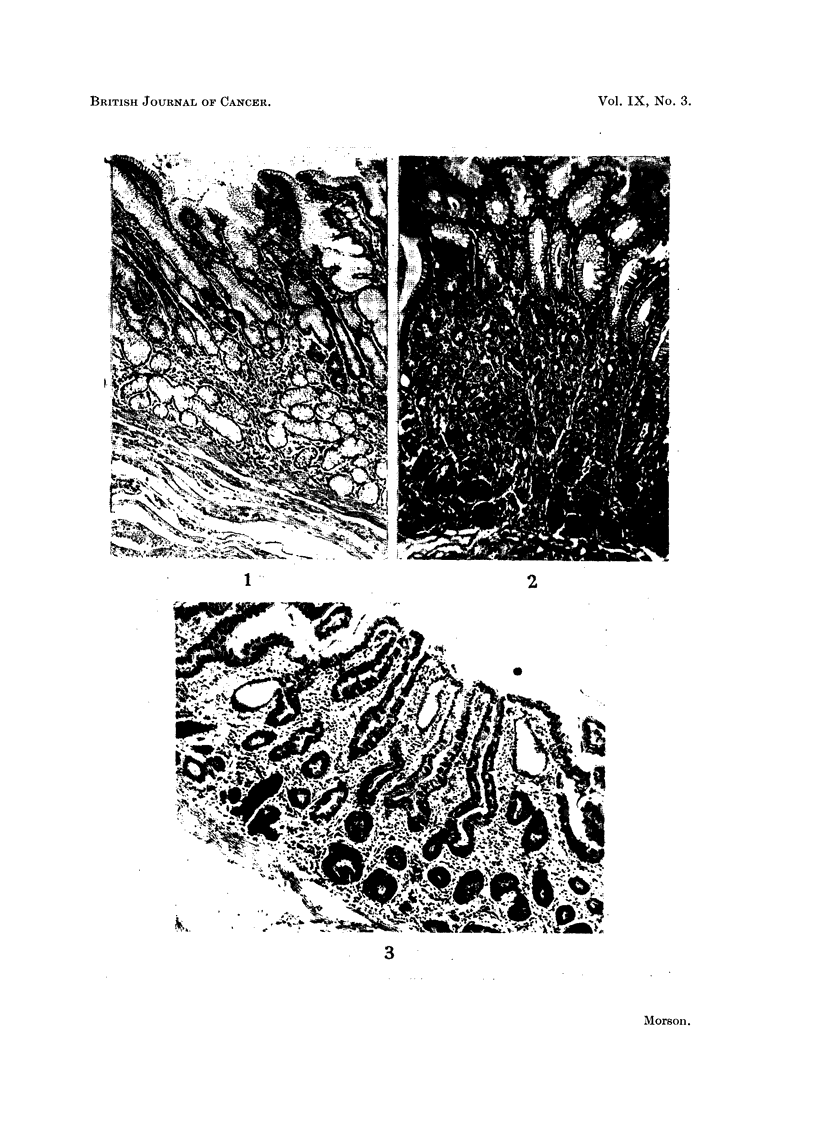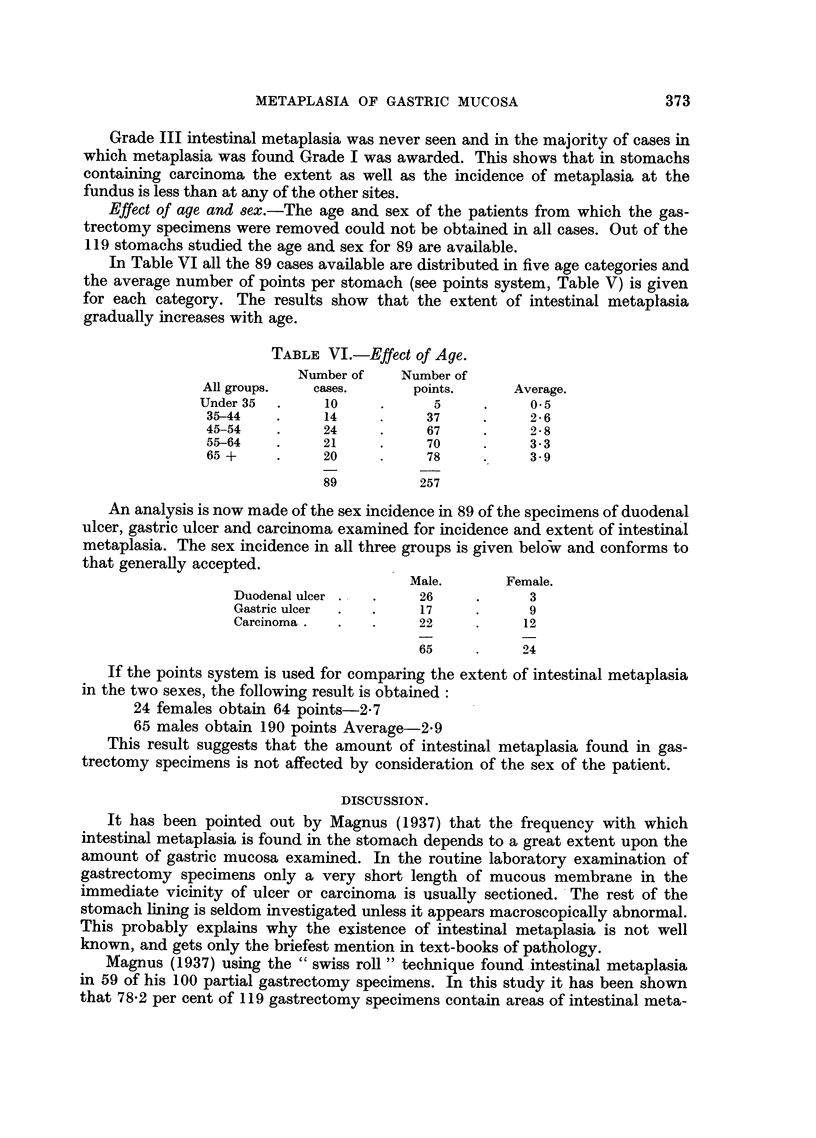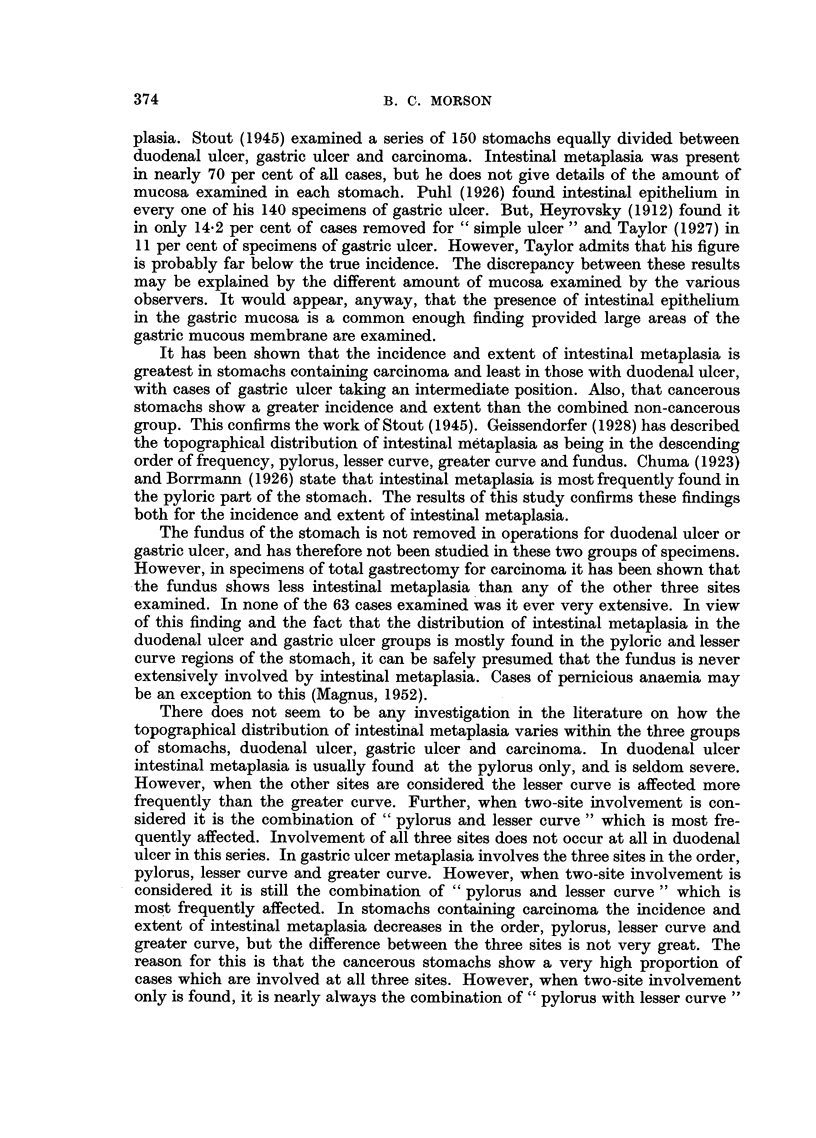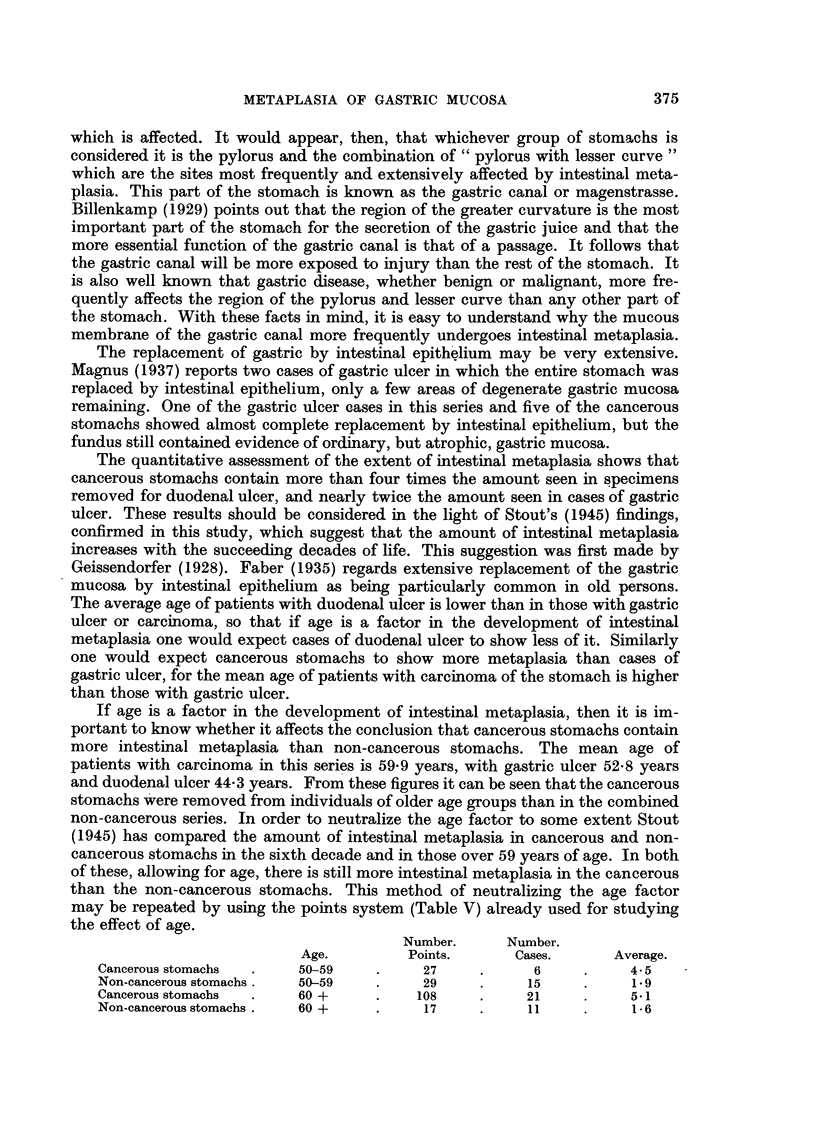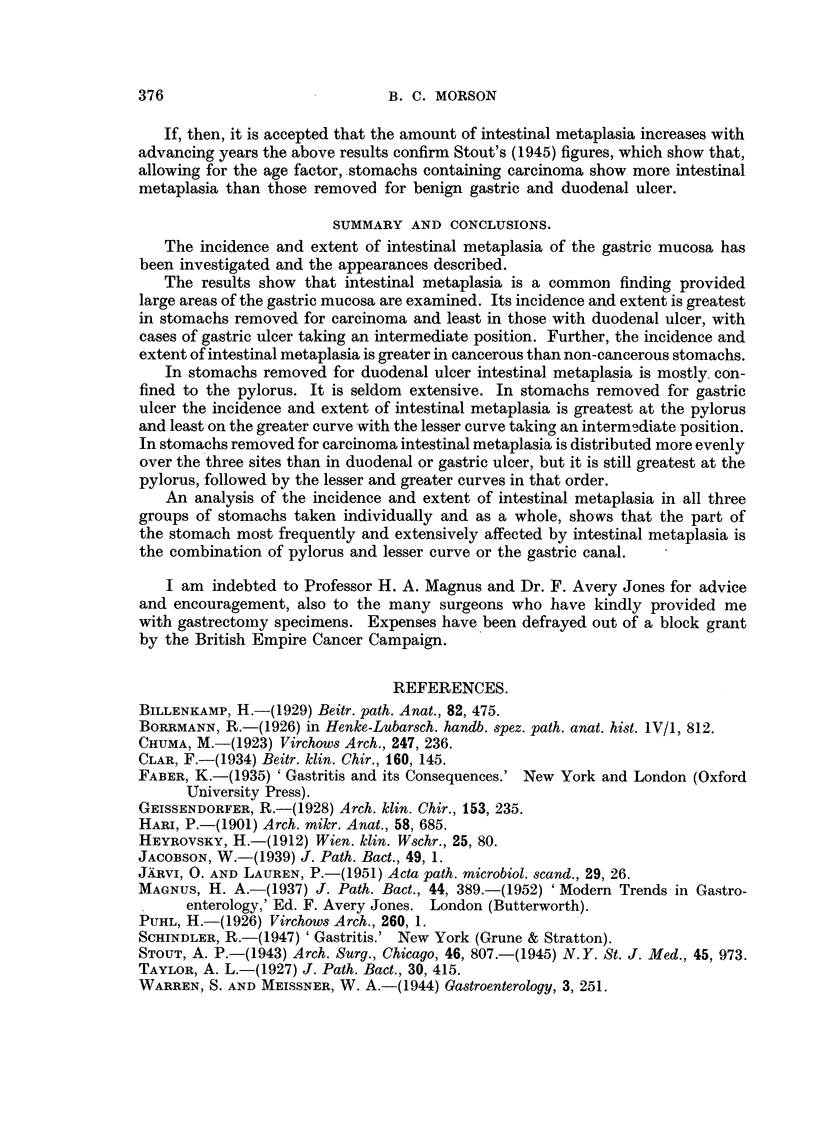# Intestinal Metaplasia of the Gastric Mucosa

**DOI:** 10.1038/bjc.1955.35

**Published:** 1955-09

**Authors:** B. C. Morson

## Abstract

**Images:**


### INTESTINAL METAPLASIA OF THE GASTRIC MUCOSA.

B. C. MORSON.

*From the Cancer Research Department, Mount Vernon Hospital, Northwood, Middlesex.*

Received for publication May 26, 1955.

DURING the routine histological examination of gastrectomy specimens removed for benign as well as malignant conditions it is not uncommon to find areas of intestinal epithelium in the gastric mucosa. The presence in the stomach lining of this type of epithelium has, in the past, been regarded as a congenital abnormality (Hari, 1901 ; Borrmann, 1926 ; Taylor, 1927 ; and Clar, 1934). However, the majority of observers in recent years favour the view that the intestinal epithelium seen in the gastric mucosa is due to a process of metaplasia in post-foetal life (Geissendorfer, 1928 ; Faber, 1935 ; Magnus, 1937 ; Stout, 1943 ; Schindler, 1947). They believe, in the words of Magnus, that " the presence of intestinal epithelium in the stomach is the result of faulty regeneration of surface epithelium in a mucosa repeatedly damaged by gastritis, and is an example of metaplasia resulting from chronic irritation" . The importance of intestinal metaplasia has been pointed out by Warren and Meissner (1944) who regard it as the chief epithelial feature of chronic gastritis.

The purpose of this paper is to draw attention to the existence of intestinal metaplasia and to describe its incidence and extent in the mucosa of stomachs removed surgically for duodenal ulcer, gastric ulcer and carcinoma.

### MATERIAL AND METHODS.

All the material used in this investigation consists of specimens removed at operations for duodenal ulcer, gastric ulcer and carcinoma. They are subdivided as follows:

| | | |
|---|---|---|
| Duodenal ulcer . . . . | 52 | specimens. |
| Gastric ulcer . . . . . | 28 | ,, |
| Carcinoma of the stomach . | 39 | ,, |
| Total . . . . . | 119 | |

Strips of stomach wall, about one centimetre wide, were taken from the entire length of the lesser and greater curvatures of every specimen ; the pylorus was included in each strip. These varied from 5 to 10 inches long. They were rolled up like a " swiss roll " (Magnus, 1937) and secured in position by a fine thread tied around the circumference. In some cases it was convenient to dissect the mucosa off the submucosa and roll up the mucous membrane by itself. By this method very long strips of gastric mucosa can be examined in one section.

*Staining techniques.*—(1) Haematoxylin and eosin. All sections were stained by this method, using Ehrlich's acid haematoxylin. Normal gastric mucus is not stained at all by this method, but the goblet cell mucus characteristic of intestinal epithelium is stained blue. (2) Southgate's modification of Mayer's muci-carmine stain. This method was only used to confirm the presence of goblet cell mucus which it stains red. It does not stain normal gastric mucus. (3) The Diazo method for staining argentaffin granules (Jacobsen, 1939). The granules are stained by Brentamine Fast red B salt (Imperial Chemical Industries). The argentaffin granules appear rust red against a yellow background. This method is quick and reliable.

On the basis of differences in the glands of which the gastric mucosa is composed the stomach may be divided for descriptive purposes into four topographical " sites ". These are, (1) the pylorus, (2) the lesser curve, (3) the greater curve, (4) the fundus.

*The pylorus.*—The pylorus is that part of the stomach which contains the pyloric glands. It may be called the pyloric " antrum " or the pyloric " region ", but for the purposes of this study it is referred to as the " pylorus ". It occupies approximately the distal quarter of the whole stomach. It is easily identified in the sections by its characteristic glands, but care must be taken not to confuse them with the areas of pseudo-pyloric metaplasia that often occur in the region of the body and fundus of the stomach. Even if intestinal metaplasia is very extensive, there is usually some evidence of the remains of typical pyloric mucosa. The pylorus is included in the " swiss roll " sections that were taken from the entire length of both curvatures of the stomach.

*The lesser curve.*—Anatomically this extends from the cardia to the pyloro-duodenal junction on the right border of the stomach. However, for the purposes of this study it is defined as the part of the anatomical lesser curve which contains the gastric glands. Its junction with pyloric mucosa is not sharply defined and the mucous membrane of the one mixes with that of the other over a short distance in the region of the incisura angularis.

*The greater curve.*—Anatomically this extends from the cardia to the pyloro-duodenal junction on the left border of the stomach. However, for the purpose of this study it is defined as that part of the greater curve which contains the gastric glands, other than the fundus. It extends somewhat further towards the pylorus than the lesser curve and almost reaches the pyloro-duodenal junction.

*The fundus.*—The fundus extends from the cardia to a point on the greater curve marked off by a plane passing horizontally through the cardiac orifice. It is only present in specimens in which total gastrectomy was performed.

" *Extent* " *of intestinal metaplasia.*—This records the amount seen within each one of the topographical sites, and is graded as follows :

| | | | |
|---|---|---|---|
| Intestinal metaplasia, Grade | I | = + | or slight extent. |
| ,, ,, ,, | II | = + + | or moderate extent. |
| ,, ,, ,, | III | = + + + | or very extensive. |

Grade I is awarded when metaplasia is present, but only in the form of scattered islands that form a very small part of the total area under consideration. However, if just one area of microscopic size is seen this grading is given, for often the process is confined to a group of three or four gastric tubules only.

Grade II is given when the islands of intestinal metaplasia have become confluent and form larger areas of metaplasia involving not more than half the total area under consideration.

Grade III intestinal metaplasia is awarded when more than half the total area under consideration is affected. Sometimes it is complete throughout the site and very little evidence of normal gastric mucosa can be found.

THE IDENTIFICATION OF INTESTINAL METAPLASIA. (Fig. 1–3)

The striking feature that distinguishes intestinal metaplasia from normal gastric epithelium is the goblet cell. This contains a droplet of mucus that is stained blue by Ehrlich's haematoxylin and red by Southgate's modification of Mayer's muci-carmine method. According to Järvi and Lauren (1951) there are two types of mucus in the human alimentary tract—gastric and intestinal. Only mucus of intestinal type stains with muci-carmine. The normal gastric mucosa is lined by tall, very regular, simple columnar epithelium and contains no goblet cells of this type. If goblet cells are seen in the gastric mucosa it can be inferred that intestinal type epithelium is present (Magnus, 1937). The surface cells of the metaplastic epithelium are columnar in type with a striated border. This feature is not seen in normal gastric epithelium but is characteristic of epithelial cells in the intestine. The goblet cells are scattered among the columnar cells and vary in number from about one goblet cell to every three or four epithelial cells up to a stage in which almost every cell in the mucosa contains the characteristic droplet of mucus. This has led, in the past, to the use of the term " goblet cell metaplasia " to describe these appearances. The deeper parts of the tubules in intestinal metaplasia are lined by columnar epithelium containing scattered goblet cells, but their nuclei are larger and more darkly staining than their counterparts in normal gastric epithelium. Goblet cells tend to be fewer in the deeper parts of the mucosa. At the bases of the metaplastic gastric glands there are features that are entirely characteristic of intestinal epithelium and are rarely found, if at all, in the normal gastric mucosa. The first of these is the Paneth cell, often seen in large numbers in areas of intestinal metaplasia, and rarely difficult to find. They are low columnar cells with basal nuclei and cytoplasm packed with coarse granules that stain brightly with eosin. In between the Paneth cells at the bases of the gastric glands argentaffin cells may be seen. These are smaller than their neighbours, but have a relatively large oval nucleus with a characteristic chromatin network. The cytoplasm contains argentaffin granules that are basal in position with the nucleus lying between them and the lumen of the tubule. They can be seen with difficulty in ordinary haematoxylin and eosin preparations, but may be clearly demonstrated by the Diazo method.

The main histological features of intestinal metaplasia have been described. They resemble, in all respects, the features of the epithelium that is found in the intestine (Magnus, 1937). However, there is a closer resemblance to the lining epithelium of the large intestine than of the small bowel. The latter shows slender villi that are not characteristic of intestinal epithelium in the gastric mucosa and goblet cells are found in much greater numbers in the large intestine.

The extent of involvement of the gastric mucous membrane varies from the conversion of a single tubule to involvement of large areas of mucosa. Uncommonly the whole lining of the stomach may be affected and it is only possible to find evidence of its gastric origin by a careful search for specialised gastric epithelial cells, such as pyloric glands. The thickness of the areas of intestinal metaplasia varies greatly. It usually conforms to that of the adjacent epithelium. In atrophic gastritis it is the same thickness as the rest of the gastric epithelium of which it is a part. However, intestinal metaplasia is not only found in association with chronic atrophic gastritis, but may also be present in other forms of gastritis. Also, in pernicious anaemia much or all of the body mucosa may be replaced by intestinal epithelium in the absence of any inflammatory lesion (Magnus, 1952).

Specialised cells of the body of the stomach may survive the process of metaplasia, and although reduced in number they are not difficult to find in most cases. On the other hand, in those cases in which large areas of the stomach mucous membrane are involved, the peptic and oxyntic cells will be entirely absent. The pyloric glands are affected in the same way, but it is usually possible to identify a particular region of the gastric mucosa that is metaplastic by surviving cells of characteristically pyloric or body type.

### THE INCIDENCE AND EXTENT OF INTESTINAL METAPLASIA

*Total incidence.*—A total of 119 stomachs removed for duodenal ulcer, gastric ulcer and carcinoma were examined for the presence of areas of intestinal metaplasia with the following result :

| | |
|---|---|
| Metaplasia present in 93 out of 119 cases = | 78·2% |
| ,, absent in 26 ,, ,, ,, ,, = | 21·8% |
| | 100·0% |

These figures show that in the examination of stomachs removed surgically one would expect to find intestinal metaplasia in the great majority of them, provided that a sufficient area of the gastric mucosa is examined ; for it must be emphasised that a positive observation includes cases in which only a very small amount of metaplasia can be seen.

*Incidence within the three groups of stomachs* (Table I).—An examination of Table I shows how the incidence of intestinal metaplasia varies within the three groups of stomachs under consideration. In stomachs containing carcinoma the incidence is 92·3 per cent, in the gastric ulcer group it is 82·1 per cent, and in the duodenal ulcer cases it is 65·4 per cent. It can be stated, therefore, that the incidence of intestinal metaplasia is greatest in cases of carcinoma and least in cases of duodenal ulcer, with cases of gastric ulcer taking an intermediate position. This table also emphasises the difference between stomachs containing carcinoma on the one hand and the combined non-cancerous group on the other ; it shows that there is, in fact, a greater incidence of intestinal metaplasia in cancerous than non-cancerous stomachs.

TABLE I.—*Incidence Within the Groups.*

| | | | | | Non-cancerous. | | | |
|---|---|---|---|---|---|---|---|---|
| Group. | – VE. | + VE. | Total. | % + VE. | – VE. | + VE. | Total. | % + VE. |
| D.U. | 18 | 34 | 52 | 65·4 | | | | |
| G.U. | 5 | 23 | 28 | 82·1 | 23 | 57 | 80 | *71·25* |
| Cancer | 3 | 36 | 39 | *92·3* | | | | |

*Incidence at the sites* (Table II).—In the stomachs removed for duodenal ulcer the pylorus is involved in nearly 60 per cent of cases, whereas the lesser curve (15·4 per cent) and the greater curve (9·6 per cent) are seldom affected. The gastric ulcer series is somewhat different ; in these the incidence at the lesser curve (57·1 per cent) and the greater curve (39·3 per cent) is relatively higher, although the pylorus still maintains its position as the site most often affected (71·4 per cent). In the gastric cancer group the order of incidence, pylorus, lesser curve and greater curve still holds good, but there is a more even distribution between the three sites. The reason for this lies in the fact, as will be shown later, that a very high proportion of cases of carcinoma shows involvement by intestinal metaplasia at all three sites (Table IIIa). It can be stated, then, that the incidence of intestinal metaplasia is greatest at the pylorus, followed by the lesser curve and greater curve in that order, whichever group of stomachs is considered.

TABLE II.—*Incidence at the Sites.*

| Site. | D.U. (%). | G.U. (%). | Carcinoma (%). |
|---|---|---|---|
| Pylorus | 59·6 | 71·4 | 87·1 |
| Lesser curve | 15·4 | 57·1 | 79·5 |
| Greater curve | 9·6 | 39·3 | 66·7 |

*The distribution of positive observations by group and site.*—Table IIIa shows that in stomachs removed for duodenal ulcer involvement of one site only is the rule (24 out of 34 cases containing intestinal metaplasia) and Table IIIb shows that this site is the pylorus. In Table IIIc it is apparent that when two-site involvement occurs in duodenal ulcer it is the combination of pylorus with lesser curve that is the most frequently affected. This combination of sites is known as the gastric canal or " magenstrasse ".

In stomachs removed for gastric ulcer two-site involvement is the most common (10 out of 23 cases containing intestinal metaplasia, Table IIIa), and Table IIIc shows that it is the combination of pylorus with lesser curve (or gastric canal) which is the most frequently affected. However, when one site only is considered (Table IIIb) it is always the pylorus which is involved. It should be

TABLE IIIa.—*Distribution of Positive Observations by Group and Site.*

| Group. | Total. | + VE | Present in: One site only. | Two sites only. | All three sites. |
|---|---|---|---|---|---|
| D.U. | 52 | 34 | 24 | 10 | 0 |
| G.U. | 28 | 23 | 6 | 10 | 7 |
| Carcinoma | 39 | 36 | 5 | 7 | 24 |

TABLE IIIb.—*One-site Involvement.*

| Site. | D.U. | G.U. | Carcinoma. | Total. |
|---|---|---|---|---|
| Pylorus | 21 | 6 | 3 | 30 |
| Lesser curve | 2 | 0 | 1 | 3 |
| Greater curve | 1 | 0 | 1 | 2 |
| Totals | 24 | 6 | 5 | 35 |

TABLE IIIc.—*Two-site involvement.*

| Site. | D.U. | G.U. | Carcinoma. | Total. |
|---|---|---|---|---|
| Pylorus and lesser curve | 6 | 6 | 6 | 18 |
| Pylorus and greater curve | 4 | 1 | 1 | 6 |
| Lesser curve and greater curve | 0 | 3 | 0 | 3 |
| Totals | 10 | 10 | 7 | 27 |

noted that there is a relatively high proportion of cases of gastric ulcer in which all three sites are affected.

In cancerous stomachs the results show the exact opposite to those found for duodenal ulcer ; namely, that three-site involvement is the most common (24 out of 36 cases containing intestinal metaplasia, Table IIIa). However, if one site only is involved it is usually the pylorus (Table IIIb), and when two-site involvement is considered it is nearly always the combination of pylorus with lesser curve (or gastric canal) which is affected.

It is the consideration of two-site involvement which is important. Apart from " pylorus with lesser curve " there are two other possible combinations of two-site involvement which can occur. They are " pylorus with greater curve " and " lesser curve with greater curve" . Table IIIc shows that these are not often found in any of the three groups of stomachs under consideration. Thus, out of a total of 119 stomachs examined, 27 show two-site involvement only, and of these the " pylorus with lesser curve " combination is found in 18 or 66·6 per cent (Table IIIc). When it is also remembered that it is nearly always the pylorus that is affected in one-site involvement (30 out of a total of 35 cases in all groups, Table IIIb) it can be seen that the part of the stomach most frequently affected by intestinal metaplasia is the gastric canal or ' magenstrasse '.

*The extent of intestinal metaplasia.* (Table IV).—In the great majority of cases of duodenal ulcer Grade I observations were made (24·4 per cent) ; these were mostly found at the pylorus (26 out of 38 Grade I observations). Only very occasionally were Grade II (2·6 per cent) or Grade III (1·3 per cent) observations made, and all of these except one were made at the pylorus. The only Grade II observation not at the pylorus is affecting the lesser curve.

In stomachs removed for gastric ulcer most of the observations are Grade I (34·5 per cent), but unlike the stomachs removed for duodenal ulcer these are more evenly distributed among the three sites. However, the order pylorus, lesser curve and greater curve is still present. The Grade II observations (7·1 per cent) are greater than in duodenal ulcer and they, too, are distributed more evenly among the three sites in the same descending order of extent. The striking feature, however, in cases of gastric ulcer is the relatively high proportion of Grade III observations (14·3 per cent) when compared with duodenal ulcer (1·3 per cent). These observations, which represent the most extensive intestinal metaplasia seen in gastric ulcer, are found most frequently in the region of the pylorus, and least frequently on the greater curve, with the lesser curve taking an intermediate position.

In the cancerous stomachs observations of all three grades are more evenly distributed among the three sites than in either gastric or duodenal ulcer. There are almost as many Grade III observations (26·5 per cent) as there are Grade I, (30·8 per cent). Also, it can be stated that the descending order of frequency for site, already demonstrated, is still the same. When cancerous stomachs are compared for extent of intestinal metaplasia with stomachs removed for duodenal and gastric ulcer the significant feature is the much greater proportion of Grade II and Grade III observations in the cancerous group. There are nearly three times as many Grade II observations in carcinomatous stomachs as there are in gastric ulcer specimens, and nearly ten times as many as there are in stomachs removed for duodenal ulcer. The greater extent of intestinal metaplasia in cancerous stomachs is emphasized by the fact that there are nearly twice the number of

Grade III observations in cancerous stomachs as in cases of gastric ulcer and more than twenty times the number in specimens removed for duodenal ulcer.

It can be stated, then, that the extent of intestinal metaplasia is greatest in stomachs containing carcinoma and least in those with duodenal ulcer, with cases of gastric ulcer taking an intermediate position. Further, the site most extensively affected by intestinal metaplasia in all cases is the pylorus, with the lesser and greater curves following in that order. However, in cases of duodenal ulcer intestinal metaplasia is mostly confined to the pylorus, and is seldom very extensive. In gastric ulcer it is greatest at the pylorus, and least on the greater curve, with the lesser curve taking an intermediate position. In cancerous stomachs, although the same descending order of frequency is still present, intestinal metaplasia is more evenly distributed over the three sites.

TABLE IVa.—*Extent of Intestinal Metaplasia by Group and Site. Duodenal Ulcer.*

| Extent. | Pylorus. | Lesser curve. | Greater curve. | Total. | Per cent. |
|---|---|---|---|---|---|
| 0 | 21 | 44 | 47 | 112 | 71·7 |
| + | 26 | 7 | 5 | 38 | 24·4 |
| ++ | 3 | 1 | 0 | 4 | 2·6 |
| +++ | 2 | 0 | 0 | 2 | 1·3 |

Total number of stomachs = 52
„ „ „ observations = 156

TABLE IVb.—*Extent of Intestinal Metaplasia by Group and Site. Gastric Ulcer.*

| Extent. | Pylorus. | Lesser curve. | Greater curve. | Total. | Per cent. |
|---|---|---|---|---|---|
| 0 | 8 | 12 | 17 | 37 | 44·1 |
| + | 11 | 10 | 8 | 29 | 34·5 |
| ++ | 3 | 2 | 1 | 6 | 7·1 |
| +++ | 6 | 4 | 2 | 12 | 14·3 |

Total number of stomachs = 28
„ „ „ observations = 84

TABLE IVc.—*Extent of Intestinal Metaplasia by Group and Site. Carcinoma.*

| Extent. | Pylorus. | Lesser curve. | Greater curve. | Total. | Per cent. |
|---|---|---|---|---|---|
| 0 | 5 | 8 | 13 | 26 | 22·2 |
| + | 12 | 13 | 11 | 36 | 30·8 |
| ++ | 8 | 7 | 9 | 24 | 20·5 |
| +++ | 14 | 11 | 6 | 31 | 26·5 |

Total number of stomachs = 39
„ „ „ observations = 117

*The points system* (Table V).—Another, and in many ways more convincing, method of demonstrating the extent of intestinal metaplasia in the three groups of stomachs is by the use of a points system. In this each stomach is awarded one point for every + observation made. For example if metaplasia is absent, obviously no points are given to that stomach ; but if a Grade II (++) observation has been made at the pylorus, and there is no metaplasia elsewhere in the stomach, then two points are awarded. This means that a maximum of nine points can be earned by a stomach which has Grade III (+++) intestinal metaplasia for each of the three sites under consideration.

The results are given in Table V. Under each group the number of stomachs are given for which a particular number of points has been awarded. The total number of points in each group are then divided by the number of stomachs. This gives the mean number of points per stomach. The results show that the mean number for carcinoma is 4·5, for gastric ulcer 2·8 and for duodenal ulcer 1·0. If the cancerous group of stomachs is then compared with the combined non-cancerous groups the figures show that the stomachs removed for carcinoma (mean number = 4·5) have more than twice the amount of intestinal metaplasia than those removed for gastric and duodenal ulcer (mean number = 1·9).

The points system confirms (1) that the amount of intestinal metaplasia is greatest in stomachs containing carcinoma and least in those with duodenal ulcer, with the gastric ulcer group taking an intermediate position, (2) that intestinal metaplasia is more extensive in cancerous than non-cancerous stomachs.

TABLE V.—*Points System.*

| | D.U. | G.U. | Carcinoma. |
|---|---|---|---|
| Total stomachs in each group | 52 | 28 | 39 |
| Total points per group | 52 | 77 | 177 |
| Mean number of points/stomach | 1·0 | 2·8 | *4·5* |
| Non-cancerous series combined | | 129 points | |
| Mean number of points/stomach | | *1·9* | |

*The incidence and extent of intestinal metaplasia at the fundus of the stomach.*—This is considered separately as observations made on the fundus can only be recorded in cases of total gastrectomy for carcinoma. In addition to the 39 stomachs previously considered a further 24 cases of total gastrectomy were available for a study of the fundus, making a total of 63 cases. Intestinal metaplasia was present in 20, or 31·8 per cent. This is the least incidence of any of the sites previously considered. In other words, in stomachs containing carcinoma there is less intestinal metaplasia at the fundus than at the pylorus, lesser curve or greater curve.

Of the 20 cases or 31·8 per cent in which intestinal metaplasia was found the extent was graded as follows :

| | | |
|---|---|---|
| Intestinal metaplasia absent | in 43 | cases. |
| ,, ,, Grade I | ,, 16 | ,, |
| ,, ,, ,, II | ,, 4 | ,, |
| ,, ,, ,, III | ,, 0 | ,, |
| | 63 | |

### EXPLANATION OF PLATES.

FIG. 1.—Normal pyloric mucosa. Haematoxylin and eosin. × 50.

FIG. 2.—Normal body mucosa. Haematoxylin and eosin. × 50.

FIG. 3.—Intestinal metaplasia of gastric mucosa. Note numerous goblet cells and contrast with the remains of three tubules lined by normal superficial gastric epithelium. Haematoxylin and eosin. × 50.

1

2

3

Morson.

Grade III intestinal metaplasia was never seen and in the majority of cases in which metaplasia was found Grade I was awarded. This shows that in stomachs containing carcinoma the extent as well as the incidence of metaplasia at the fundus is less than at any of the other sites.

*Effect of age and sex.*—The age and sex of the patients from which the gastrectomy specimens were removed could not be obtained in all cases. Out of the 119 stomachs studied the age and sex for 89 are available.

In Table VI all the 89 cases available are distributed in five age categories and the average number of points per stomach (see points system, Table V) is given for each category. The results show that the extent of intestinal metaplasia gradually increases with age.

TABLE VI.—*Effect of Age.*

| All groups. | Number of cases. | Number of points. | Average. |
|---|---|---|---|
| Under 35 | 10 | 5 | 0·5 |
| 35–44 | 14 | 37 | 2·6 |
| 45–54 | 24 | 67 | 2·8 |
| 55–64 | 21 | 70 | 3·3 |
| 65 + | 20 | 78 | 3·9 |
| | 89 | 257 | |

An analysis is now made of the sex incidence in 89 of the specimens of duodenal ulcer, gastric ulcer and carcinoma examined for incidence and extent of intestinal metaplasia. The sex incidence in all three groups is given below and conforms to that generally accepted.

| | Male. | Female. |
|---|---|---|
| Duodenal ulcer | 26 | 3 |
| Gastric ulcer | 17 | 9 |
| Carcinoma | 22 | 12 |
| | 65 | 24 |

If the points system is used for comparing the extent of intestinal metaplasia in the two sexes, the following result is obtained :

24 females obtain 64 points—2·7

65 males obtain 190 points Average—2·9

This result suggests that the amount of intestinal metaplasia found in gastrectomy specimens is not affected by consideration of the sex of the patient.

### DISCUSSION.

It has been pointed out by Magnus (1937) that the frequency with which intestinal metaplasia is found in the stomach depends to a great extent upon the amount of gastric mucosa examined. In the routine laboratory examination of gastrectomy specimens only a very short length of mucous membrane in the immediate vicinity of ulcer or carcinoma is usually sectioned. The rest of the stomach lining is seldom investigated unless it appears macroscopically abnormal. This probably explains why the existence of intestinal metaplasia is not well known, and gets only the briefest mention in text-books of pathology.

Magnus (1937) using the " swiss roll " technique found intestinal metaplasia in 59 of his 100 partial gastrectomy specimens. In this study it has been shown that 78·2 per cent of 119 gastrectomy specimens contain areas of intestinal metaplasia. Stout (1945) examined a series of 150 stomachs equally divided between duodenal ulcer, gastric ulcer and carcinoma. Intestinal metaplasia was present in nearly 70 per cent of all cases, but he does not give details of the amount of mucosa examined in each stomach. Puhl (1926) found intestinal epithelium in every one of his 140 specimens of gastric ulcer. But, Heyrovsky (1912) found it in only 14·2 per cent of cases removed for " simple ulcer " and Taylor (1927) in 11 per cent of specimens of gastric ulcer. However, Taylor admits that his figure is probably far below the true incidence. The discrepancy between these results may be explained by the different amount of mucosa examined by the various observers. It would appear, anyway, that the presence of intestinal epithelium in the gastric mucosa is a common enough finding provided large areas of the gastric mucous membrane are examined.

It has been shown that the incidence and extent of intestinal metaplasia is greatest in stomachs containing carcinoma and least in those with duodenal ulcer, with cases of gastric ulcer taking an intermediate position. Also, that cancerous stomachs show a greater incidence and extent than the combined non-cancerous group. This confirms the work of Stout (1945). Geissendorfer (1928) has described the topographical distribution of intestinal metaplasia as being in the descending order of frequency, pylorus, lesser curve, greater curve and fundus. Chuma (1923) and Borrmann (1926) state that intestinal metaplasia is most frequently found in the pyloric part of the stomach. The results of this study confirms these findings both for the incidence and extent of intestinal metaplasia.

The fundus of the stomach is not removed in operations for duodenal ulcer or gastric ulcer, and has therefore not been studied in these two groups of specimens. However, in specimens of total gastrectomy for carcinoma it has been shown that the fundus shows less intestinal metaplasia than any of the other three sites examined. In none of the 63 cases examined was it ever very extensive. In view of this finding and the fact that the distribution of intestinal metaplasia in the duodenal ulcer and gastric ulcer groups is mostly found in the pyloric and lesser curve regions of the stomach, it can be safely presumed that the fundus is never extensively involved by intestinal metaplasia. Cases of pernicious anaemia may be an exception to this (Magnus, 1952).

There does not seem to be any investigation in the literature on how the topographical distribution of intestinal metaplasia varies within the three groups of stomachs, duodenal ulcer, gastric ulcer and carcinoma. In duodenal ulcer intestinal metaplasia is usually found at the pylorus only, and is seldom severe. However, when the other sites are considered the lesser curve is affected more frequently than the greater curve. Further, when two-site involvement is considered it is the combination of " pylorus and lesser curve " which is most frequently affected. Involvement of all three sites does not occur at all in duodenal ulcer in this series. In gastric ulcer metaplasia involves the three sites in the order, pylorus, lesser curve and greater curve. However, when two-site involvement is considered it is still the combination of " pylorus and lesser curve " which is most frequently affected. In stomachs containing carcinoma the incidence and extent of intestinal metaplasia decreases in the order, pylorus, lesser curve and greater curve, but the difference between the three sites is not very great. The reason for this is that the cancerous stomachs show a very high proportion of cases which are involved at all three sites. However, when two-site involvement only is found, it is nearly always the combination of " pylorus with lesser curve "

which is affected. It would appear, then, that whichever group of stomachs is considered it is the pylorus and the combination of " pylorus with lesser curve " which are the sites most frequently and extensively affected by intestinal metaplasia. This part of the stomach is known as the gastric canal or magenstrasse. Billenkamp (1929) points out that the region of the greater curvature is the most important part of the stomach for the secretion of the gastric juice and that the more essential function of the gastric canal is that of a passage. It follows that the gastric canal will be more exposed to injury than the rest of the stomach. It is also well known that gastric disease, whether benign or malignant, more frequently affects the region of the pylorus and lesser curve than any other part of the stomach. With these facts in mind, it is easy to understand why the mucous membrane of the gastric canal more frequently undergoes intestinal metaplasia.

The replacement of gastric by intestinal epithelium may be very extensive. Magnus (1937) reports two cases of gastric ulcer in which the entire stomach was replaced by intestinal epithelium, only a few areas of degenerate gastric mucosa remaining. One of the gastric ulcer cases in this series and five of the cancerous stomachs showed almost complete replacement by intestinal epithelium, but the fundus still contained evidence of ordinary, but atrophic, gastric mucosa.

The quantitative assessment of the extent of intestinal metaplasia shows that cancerous stomachs contain more than four times the amount seen in specimens removed for duodenal ulcer, and nearly twice the amount seen in cases of gastric ulcer. These results should be considered in the light of Stout's (1945) findings, confirmed in this study, which suggest that the amount of intestinal metaplasia increases with the succeeding decades of life. This suggestion was first made by Geissendorfer (1928). Faber (1935) regards extensive replacement of the gastric mucosa by intestinal epithelium as being particularly common in old persons. The average age of patients with duodenal ulcer is lower than in those with gastric ulcer or carcinoma, so that if age is a factor in the development of intestinal metaplasia one would expect cases of duodenal ulcer to show less of it. Similarly one would expect cancerous stomachs to show more metaplasia than cases of gastric ulcer, for the mean age of patients with carcinoma of the stomach is higher than those with gastric ulcer.

If age is a factor in the development of intestinal metaplasia, then it is important to know whether it affects the conclusion that cancerous stomachs contain more intestinal metaplasia than non-cancerous stomachs. The mean age of patients with carcinoma in this series is 59·9 years, with gastric ulcer 52·8 years and duodenal ulcer 44·3 years. From these figures it can be seen that the cancerous stomachs were removed from individuals of older age groups than in the combined non-cancerous series. In order to neutralize the age factor to some extent Stout (1945) has compared the amount of intestinal metaplasia in cancerous and non-cancerous stomachs in the sixth decade and in those over 59 years of age. In both of these, allowing for age, there is still more intestinal metaplasia in the cancerous than the non-cancerous stomachs. This method of neutralizing the age factor may be repeated by using the points system (Table V) already used for studying the effect of age.

| | Age. | Number. Points. | Number. Cases. | Average. |
|---|---|---|---|---|
| Cancerous stomachs | 50–59 | 27 | 6 | 4·5 |
| Non-cancerous stomachs | 50–59 | 29 | 15 | 1·9 |
| Cancerous stomachs | 60 + | 108 | 21 | 5·1 |
| Non-cancerous stomachs | 60 + | 17 | 11 | 1·6 |

If, then, it is accepted that the amount of intestinal metaplasia increases with advancing years the above results confirm Stout's (1945) figures, which show that, allowing for the age factor, stomachs containing carcinoma show more intestinal metaplasia than those removed for benign gastric and duodenal ulcer.

### SUMMARY AND CONCLUSIONS.

The incidence and extent of intestinal metaplasia of the gastric mucosa has been investigated and the appearances described.

The results show that intestinal metaplasia is a common finding provided large areas of the gastric mucosa are examined. Its incidence and extent is greatest in stomachs removed for carcinoma and least in those with duodenal ulcer, with cases of gastric ulcer taking an intermediate position. Further, the incidence and extent of intestinal metaplasia is greater in cancerous than non-cancerous stomachs.

In stomachs removed for duodenal ulcer intestinal metaplasia is mostly confined to the pylorus. It is seldom extensive. In stomachs removed for gastric ulcer the incidence and extent of intestinal metaplasia is greatest at the pylorus and least on the greater curve with the lesser curve taking an intermediate position. In stomachs removed for carcinoma intestinal metaplasia is distributed more evenly over the three sites than in duodenal or gastric ulcer, but it is still greatest at the pylorus, followed by the lesser and greater curves in that order.

An analysis of the incidence and extent of intestinal metaplasia in all three groups of stomachs taken individually and as a whole, shows that the part of the stomach most frequently and extensively affected by intestinal metaplasia is the combination of pylorus and lesser curve or the gastric canal.

I am indebted to Professor H. A. Magnus and Dr. F. Avery Jones for advice and encouragement, also to the many surgeons who have kindly provided me with gastrectomy specimens. Expenses have been defrayed out of a block grant by the British Empire Cancer Campaign.

### REFERENCES.

BILLENKAMP, H.—(1929) *Beitr. path. Anat.*, **82**, 475.
BORRMANN, R.—(1926) in *Henke-Lubarsch. handb. spez. path. anat. hist.* IV/1, 812.
CHUMA, M.—(1923) *Virchows Arch.*, **247**, 236.
CLAR, F.—(1934) *Beitr. klin. Chir.*, **160**, 145.
FABER, K.—(1935) 'Gastritis and its Consequences.' New York and London (Oxford University Press).
GEISSENDORFER, R.—(1928) *Arch. klin. Chir.*, **153**, 235.
HARI, P.—(1901) *Arch. mikr. Anat.*, **58**, 685.
HEYROVSKY, H.—(1912) *Wien. klin. Wschr.*, **25**, 80.
JACOBSON, W.—(1939) *J. Path. Bact.*, **49**, 1.
JÄRVI, O. AND LAUREN, P.—(1951) *Acta path. microbiol. scand.*, **29**, 26.
MAGNUS, H. A.—(1937) *J. Path. Bact.*, **44**, 389.—(1952) 'Modern Trends in Gastroenterology,' Ed. F. Avery Jones. London (Butterworth).
PUHL, H.—(1926) *Virchows Arch.*, **260**, 1.
SCHINDLER, R.—(1947) 'Gastritis.' New York (Grune & Stratton).
STOUT, A. P.—(1943) *Arch. Surg., Chicago*, **46**, 807.—(1945) *N.Y. St. J. Med.*, **45**, 973.
TAYLOR, A. L.—(1927) *J. Path. Bact.*, **30**, 415.
WARREN, S. AND MEISSNER, W. A.—(1944) *Gastroenterology*, **3**, 251.